# Neurodevelopmental Profile of a 4.5-Year-Old Girl with Tetrasomy X

**DOI:** 10.3390/pediatric18020040

**Published:** 2026-03-09

**Authors:** Maša Marisavljević, Nina Stanojević, Ivana Bogavac, Ivana Milanović, Slavica Maksimović, Silvana Punišić, Jelena Đorđević

**Affiliations:** 1 Cognitive Neuroscience Department, Research and Development Institute “Life Activities Advancement Institute”, 11000 Belgrade, Serbia; n.stanojevic@add-for-life.com (N.S.); i.bogavac@add-for-life.com (I.B.); i.milanovic@add-for-life.com (I.M.); s.maksimovic@add-for-life.com (S.M.); s.punisic@add-for-life.com (S.P.); 2 Department of Speech, Language and Hearing Sciences, Institute for Experimental Phonetics and Speech Pathology, 11000 Belgrade, Serbia; 3 Clinic for Neurology and Psychiatry for Children and Adolescents, 11000 Belgrade, Serbia; jelena.djordjevic@medf.kg.ac.rs; 4 Department of Psychiatry, Faculty of Medical Sciences, University of Kragujevac, 34000 Kragujevac, Serbia

**Keywords:** Tetrasomy X, 48, XXXX, sex chromosome aneuploidy, neurodevelopmental profile

## Abstract

**Background**: Tetrasomy X (48, XXXX) is an extremely rare sex chromosome aneuploidy characterized by highly variable phenotypic manifestations. It includes various medical issues, a wide range of developmental delays, and neurocognitive deficits. **Methods**: The present case report provides a comprehensive neurodevelopmental profile of a 4.5-year-old girl with Tetrasomy X, with the aim of contributing to phenotype delineation, exploring genotype–phenotype associations, and emphasizing the importance of early, targeted intervention. A multidisciplinary assessment was conducted, encompassing cognitive, speech–language, motor, sensory, adaptive, and socioemotional functioning, using a battery of standardized and culturally adapted instruments. **Results**: Results revealed borderline intellectual functioning and mild global developmental delay, with marked intra-individual variability across domains. Motor development was significantly delayed and speech and language assessment demonstrated a pronounced receptive–expressive discrepancy. Sensory processing evaluation revealed a pattern of global sensory under-responsiveness, representing a novel and underreported feature in Tetrasomy X. Adaptive functioning was uneven, with relative strengths in daily living skills and weaknesses in motor abilities. **Conclusions**: This detailed early developmental characterization highlights the heterogeneity of Tetrasomy X and challenges some of the previous assumptions. The findings underscore the necessity of individualized, multidisciplinary assessment and early intervention to optimize developmental outcomes and quality of life in affected individuals.

## 1. Introduction

Tetrasomy X (48, XXXX) is a rare sex chromosome aneuploidy (SCA) characterized by the presence of two additional X chromosomes in females, resulting in a 48, XXXX karyotype. First described in 1961, Tetrasomy X remains an exceptionally uncommon chromosomal condition. To date, approximately 100 cases have been reported worldwide, with around 60 individuals described in the scientific literature [1,2,3,4]. Roughly one-third of these reported cases involve adolescent or adult females [2,5]. The precise prevalence of Tetrasomy X remains unknown. The clinical phenotype of individuals with Tetrasomy X is characterized by a spectrum of medical and neurodevelopmental manifestations, including somatic anomalies, growth disturbances, and variable degrees of motor, cognitive, and speech–language delay [1,4,5,6,7,8,9,10,11,12,13,14,15,16]

When it comes to developmental outcomes, low muscle tone and poor sensorimotor coordination are frequently reported, contributing to delayed motor skill acquisition, including the late onset of independent walking [17,18,19]. In addition, significantly impaired visual–motor skills, particularly in graphomotor and perceptual domains, as well as mildly reduced visual perception abilities, have been documented [14]. Although studies from the previous century generally indicated that Tetrasomy X is accompanied by intellectual disability [5,8,20,21,22,23,24], more recent research suggests IQ scores ranging from below average to average, with reported values between 30 and 90 [1,6,11,14,25]. In other words, children with Tetrasomy X often present with reduced cognitive abilities, with 30–50% meeting criteria for mild intellectual disability [26]. Clinical observations further indicate that IQ tends to decrease by approximately 10–15 points for each additional X chromosome [1,2]. Speech and language impairments are among the most consistently reported challenges in individuals with Tetrasomy X [6,11,13,14,27,28,29,30,31,32,33,34]. The most frequently described deficits include delayed language acquisition [28], reduced expressive language, and speech sound disorders as well as apraxia and dyspraxia [14,27,32,34]. Interestingly, research indicates a dose-dependent effect of the number of X chromosomes on language functioning, with additional X chromosomes associated with more pronounced verbal fluency impairments [35]. Specifically, phonemic fluency appears to be more affected than semantic fluency. Notably, there are reports of women with normal intellectual functioning who nevertheless present with severe articulation difficulties and markedly reduced speech skills [28].

In addition, behavioral phenotypes are highly variable: some individuals are friendly, cooperative, and socially engaged, whereas others may exhibit social inappropriateness, aggression, and emotional lability [1,5,14,33,36,37]. Language-based learning deficits can further hinder social development, sometimes leading to frustration and oppositional behaviors [38]. Other commonly reported mental health concerns include anxiety, depression, oversensitivity to sensory stimuli, and an increased prevalence of autism spectrum disorder (ASD) [39,40,41]. Some studies also report higher frequencies of schizophrenia-like symptoms, epilepsy, and hypersomnia [1,42,43,44].

The limited number and heterogeneity of reported cases hinder understanding of the Tetrasomy X phenotype. Existing studies show marked phenotypic variability and no distinctive features, complicating clinical recognition and likely contributing to underdiagnosis. After an initial surge in interest following its discovery, research declined due to the absence of a consistent clinical profile, with only modest renewed attention in recent years. Most studies have focused on general developmental deviations without providing detailed neurocognitive, speech–language, and psychological profiles. Additionaly, frequent grouping of SCAs limits identification of Tetrasomy X-specific characteristics. The present case report aims to address these gaps by providing a detailed cognitive, speech–language, motor, sensory, adaptive, and socioemotional profile of a 4.5-year-old girl with Tetrasomy X. To our knowledge, this is the first report to make available an in-depth developmental characterization at this early age. Our aims are threefold: (1) to contribute a carefully characterized phenotype to the limited body of literature on Tetrasomy X; (2) to explore potential genotype–phenotype correlations; and (3) to emphasize the importance of precise diagnostic evaluation to enable timely and targeted intervention.

## 2. Case Presentation

### 2.1. Case Report

**Figure 1:**
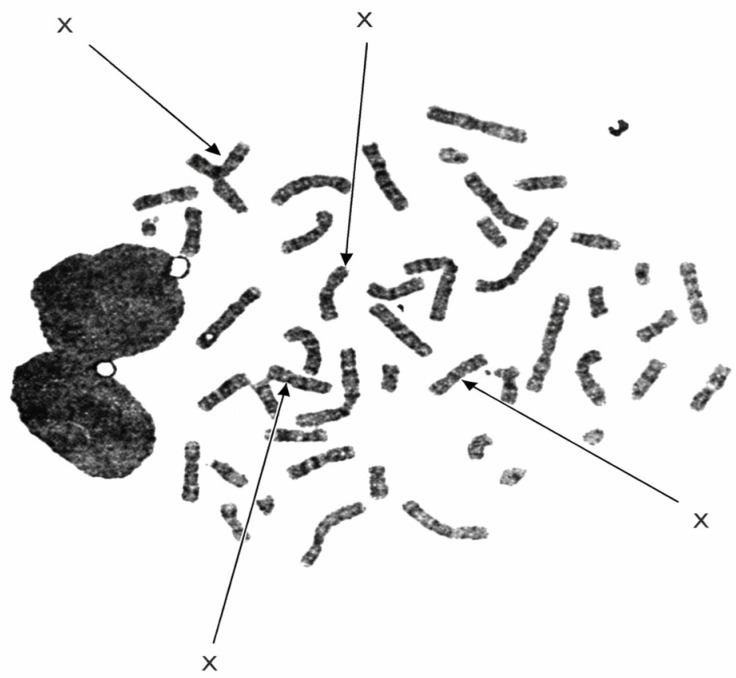
Representative metaphase spread showing four X chromosomes (48, XXXX) identified by GTG banding. An arranged karyotype was not available from the original clinical laboratory report.

The girl described is the second of two children. At her birth, the father was 41 years old and the mother was 39. The parents are non-consanguineous and in good health, with no significant medical history on either side; however, a positive family history of epilepsy was reported in the extended family. There is no history of miscarriage. She has an older brother, aged 8, who is also in good health. The family resides in a town in the Republic of Serbia. The child is a native Serbian speaker, growing up in a monolingual household. The child was born from the second pregnancy at full term (40 + 5 weeks of gestation) via Caesarean section. Apgar scores were 8 at first minute and 9 at five minutes after birth. Birth measurements were: weight 3500 g, length 52 cm, and occipitofrontal circumference 36 cm. Due to signs of a possible perinatal infection, she was transferred to the Neonatology Department for observation and appropriate treatment. At birth, she was eupneic, eucardiac, febrile, and exhibited a pale skin tone. Early postnatal complications included rhinitis and a urinary tract infection, for which she received antibiotic therapy. Early gross motor development was significantly delayed. The child was diagnosed with hypotonia and began walking independently at 2.5 years of age. Language development was also significantly delayed. Early prelinguistic milestones, such as cooing and babbling, appeared around 8–9 months, later than typically expected. While first meaningful words emerged around 12 months, two-word combinations were absent by age two, indicating delayed expressive language development. Clinical genetic evaluation at 7 months of age revealed distinctive facial dysmorphic features, including a broad nasal bridge, epicanthal folds, a single transverse palmar crease on the right hand, and synophrys (joined eyebrows). Consequently, the family initiated genetic testing. At 18 months, cytogenetic analysis of peripheral blood lymphocytes demonstrated a female karyotype with 48 chromosomes. Tetrasomy X was identified by G-banding using trypsin and Giemsa (GTG banding technique) in all 16 analyzed metaphases. Based on these consistent findings, the child was diagnosed with Tetrasomy X (see Figure 1)

Further consultations included a physiatrist and a neurologist. Diagnoses included hypotonia and ankle joint instability. Clinical examination further revealed planovalgus foot deformity. Based on these findings, the use of orthopedic footwear was recommended to support joint stability and improve gait and she had physiotherapy. Audiological assessments, including Transient Evoked Otoacoustic Emissions (TEOAEs) and Brainstem Evoked Response Audiometry (BERA), demonstrated normal hearing function. She is under regular ophthalmological follow-up. On presentation, her bilateral visual acuity was 0.7 OU. Cycloplegic refraction revealed myopic astigmatism: OD −1.50 D sph/−2.00 D cyl × 175°; OS −0.50 D sph/−0.75 D cyl × 20°. The cover test, Hirschberg test, and ocular motility were all within normal limits. Spectacle correction was prescribed according to the refractive error, and she currently wears corrective eyeglasses. Due to a history of recurrent urinary tract infections, she was evaluated by a nephrologist at the age of 3 years, who identified dilation of the renal collecting system. A diagnosis of acute nephritis was established. Dimercaptosuccinic acid scan (DMSA) analysis demonstrated a moderate, diffuse reduction in tubular function of the left kidney, predominantly affecting the upper pole. At her most recent endocrinology visit (age 4), laboratory testing revealed IGF-1 levels below the expected range for her age and sex (72 ng/mL), with normal thyroid function tests (TSH: 1.92 mIU/mL, FT4: 15.57 pmol/L). Bone age, assessed by radiography, was consistent with chronological age. She has no history of emotional or behavioral difficulties and is not currently receiving pharmacological treatment.

At 4.5 years of age, the child was referred to the Institute for Experimental Phonetics and Speech Pathology (IEPSP) in Belgrade, Serbia, for comprehensive speech–language intervention and advanced diagnostics. Extensive cognitive and linguistic assessments were performed, after which individualized therapy was initiated in accordance with Kostić’s Selective Auditory Filter Amplifier (KSAFA) framework, recognized for its evidence-based efficacy in improving auditory and speech–language processing [45,46,47]. Results of assessment are presented below.

### 2.2. Data Collection

The comprehensive assessment was conducted over three morning sessions on separate days, to optimize attention and minimize fatigue. All sessions was done in a quiet, distraction-free room with minimal visual stimuli. The neurodevelopmental assessment was done by an experienced child psychologist and a speech and language pathologist, each with over 10 years of clinical experience.

The psychological evaluation included the administration of the following standardized instruments: the New Belgrade Binet–Simon Scale (NBBS) [48], the Čuturić Developmental Test (RTČ-P) [49], and the Vineland Adaptive Behavior Scales, second edition: parent/caregiver rating form (VABS-II) [50].

The speech and language evaluation included the administration of the three standardized instruments: the Peabody Picture Vocabulary Test, third edition—Croatian version (PPVT-III-HR) [51] and the New Reynell Developmental Language Scales, Serbian version (NRDLS-SR) [52], as well as one test used in clinical practice for the last decade in Serbia: the Global Articulation Test (GAT) [53].

In addition, her parents were provided with the following questionnaires to complete at home: the Sensory Profile (SP-2) [54], the Gilliam Autism Rating Scale, third edition (GARS-3) [55], and the Children’s Communication Checklist—Second Edition (CCC-2) [56].

A detailed description of administred instruments can be found in the Supplementary Materials.

## 3. Results

### 3.1. General Observation

The child walks independently but demonstrates clumsiness when running or navigating stairs. She does not ride a bicycle or scooter. She is able to jump vertically, but cannot jump forward or on one leg. She can kick, catch, and throw larger balls. Regarding her fine motor skills, she exhibits difficulties with fine manipulation of objects. She can assemble shape sorters and puzzles, and can classify objects by color. She demonstrates challenges in maintaining a strong hand grip. She is beginning to construct three-dimensional structures using blocks and uses rotational wrist movements. Graphomotor skills are consistent with an undifferentiated pencil grip, and her drawings are limited to lines and circles. Functional lateralization indicates right-handedness.

The child responds adequately to both low- and high-frequency sounds and can localize them successfully. She demonstrates comprehension of simple verbal commands involving a single task, as well as two-step instructions when familiar from prior experience. She understands basic and some more complex questions, particularly when they relate to her personal experience, and is capable of comprehending certain morphosyntactically complex questions when contextual support is provided. Gesture development has progressed typically. She uses facial expressions, gestures, and body language effectively. Her nonverbal communication skills are well developed and age-appropriate, allowing her to express emotions and needs successfully without words. The child demonstrates a marked delay in expressive language. In spontaneous speech, she primarily produces simple to minimally expanded sentences that are largely agrammatical. Morphological markers for gender, number, and tense are frequently omitted, and auxiliary words and prepositions are often absent. Her vocabulary is limited, consisting mainly of nouns, verbs, and adjectives. Pronounced phonetic–phonological deficits reduce overall speech intelligibility. Speech is generally comprehensible to unfamiliar listeners only with contextual support, though it serves a functional communicative purpose. She can ask questions beginning with “who,” “what,” and “where,” but not “when,” “how,” or “why.” She knows her first and last name, as well as the names of close family members and familiar individuals.

Sleep and appetite are regular. She feeds herself independently, including appropriate use of utensils, and is fairly independent in dressing and undressing. She requires assistance with tasks such as fastening zippers and buttons, reflecting her motor difficulties. Anal sphincter control and daytime urinary continence were achieved by age 3; however, primary nocturnal enuresis persists. Personal hygiene requires supervision.

The child maintains good and consistent eye contact. She is warm, cheerful, and frequently smiling, with a lively personality. She may occasionally appear somewhat withdrawn, likely due to awareness of her speech limitations, yet overall presents as a warm and engaging child. She actively participates in imaginative play with peers and initiates social contact; however, more complex peer interactions are limited by her language difficulties. She is integrated into kindergarten with support from a personal assistant.

The mother reports prior auditory sensitivities. Currently, tactile sensitivities are noted, particularly aversion to hair washing and the sensation of hair touching her face.

### 3.2. Test Results

#### 3.2.1. Cognitive Profile

**Figure 2:**
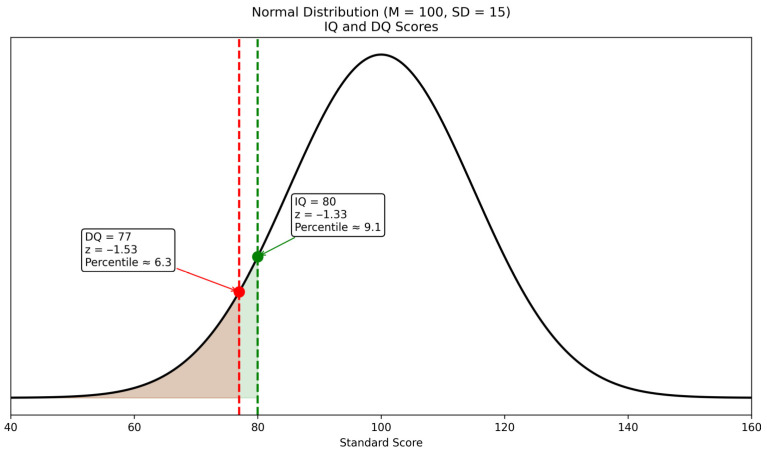
Intellectual and developmental functioning.

Figure 2 presents the results of the NBBS and the RTČ-P, indicating functioning within the borderline to low-average intellectual range, along with delayed attainment of age-appropriate developmental milestones.

#### 3.2.2. Speech and Language Profile

Her speech and language evaluation indicates dysharmonic profile. On the PPVT-III-HR, the child achieved a standardized score of 109, placing her in the above-average range. This corresponds to a receptive vocabulary age equivalent of 5.02 years, indicating that her receptive vocabulary skills are slightly advanced relative to her chronological age of 4.5 years.

**Figure 3:**
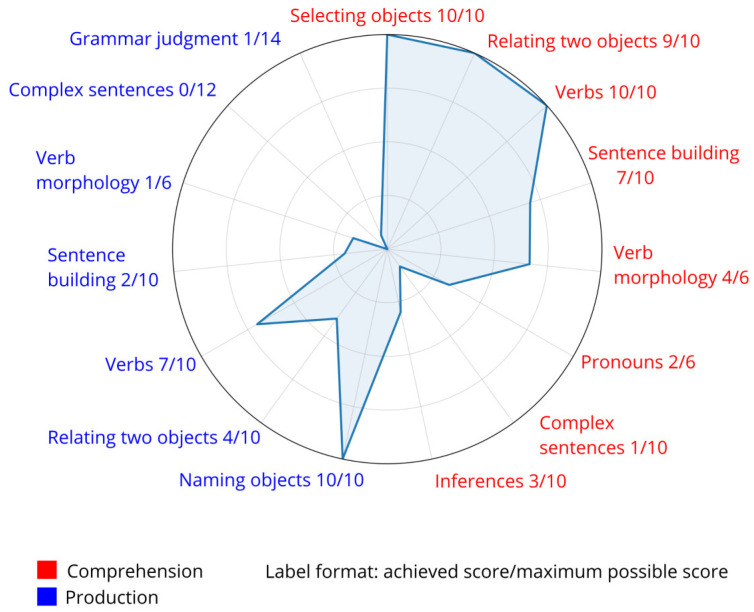
Reynell language profile.

On the NRDLS-SR, she obtained a standardized score of 46 on the Language Comprehension Scale, corresponding to a developmental age equivalent of 3.00 years, and a score of 25 on the Language Production Scale, corresponding to a developmental age equivalent of 2.09 years. Her composite language score was 69, placing her performance in the below-average range compared to same-age peers. Figure 3 shows a radar plot of the Reynell Language Profile with an uneven language profile, relatively stronger performance in single-word production (particularly nouns and verbs), and markedly weaker performance in higher-order grammatical structures, complex sentence processing, and inferential language skills.

**Figure 4:**
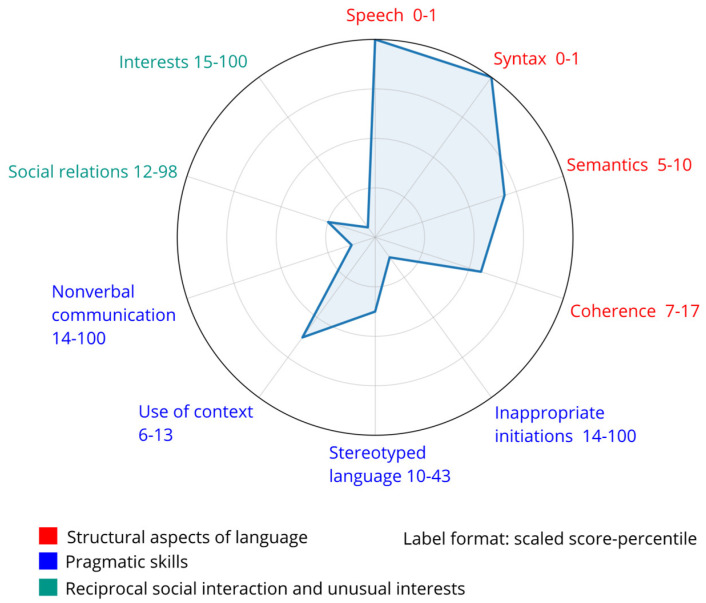
Child’s communication profile (inverted radar; higher scores closer to the center).

On the CCC-2, the child obtained a General Communication Composite (GCC) score of 83, corresponding to the 48th percentile. This indicates that her overall communication abilities are slightly below age expectations; in other words, she performed better than 48% of children in the normative sample but below 52% of her peers. Her SIDC score was +43. A positive SIDC score suggests that her pragmatic skills are relatively stronger than her core language abilities. Analysis of individual subscales is presented in the Figure 4.

On the GAT and through general speech observation, the child was found to exhibit marked nasality and muffled resonance, with pitch ranging from extremely high to rough phonation, the latter accompanied by reduced audibility. Excluding nasality as a primary vocal feature, performance on the GAT is shown in the Table 1.

#### 3.2.3. Sensory Profile

**Figure 5:**
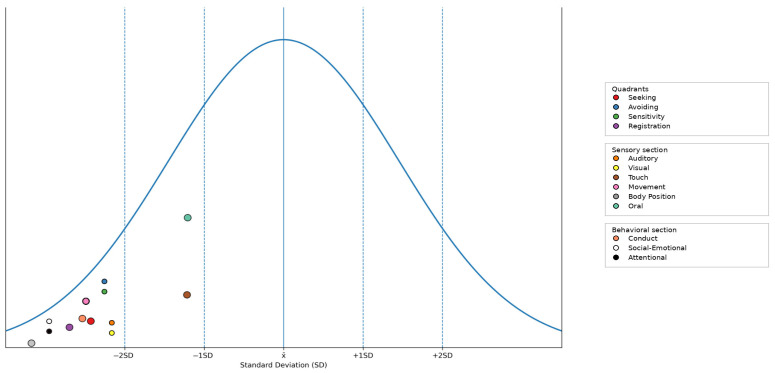
Sensory profile results.

Results obtained on the SP-2 indicate that the child’s sensory processing deviates significantly from typical developmental norms (Figure 5). Scores consistently fell within the –1 SD to –2 SD range across the quadrant, sensory, and behavioral sections of the profile. The child’s sensory processing patterns were characterized as occurring “less” or “much less” than others, indicating a reduced frequency of sensory-related behaviors compared to same-age peers.

#### 3.2.4. Profile of Adaptive Functioning

**Figure 6:**
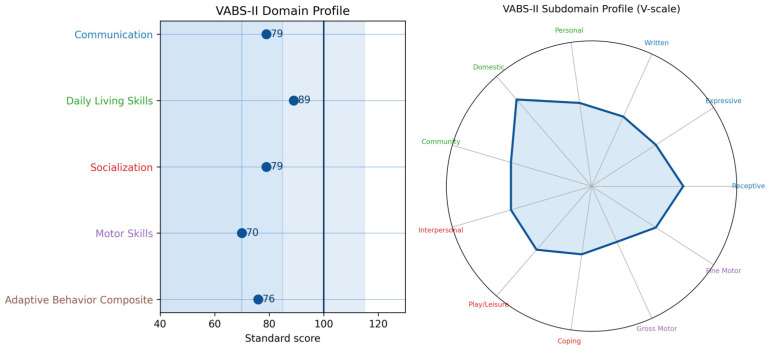
Child’s profile of adaptive functioning.

Results obtained on the VABS-II indicate that the child’s overall level of adaptive functioning falls within the moderately low range (Figure 6), with notable variability across different areas of functioning.

The Maladaptive Behavior Index (including both internalizing and externalizing behaviors) falls within the average range, suggesting that the child’s emotional and behavioral functioning is broadly within age-appropriate expectations. These findings indicate the absence of clinically significant levels of behavioral difficulties, meaning that no major concerns regarding emotional regulation, aggression, withdrawal, or other maladaptive behaviors were observed. On the GARS-3, the child obtained an Autism Index Score of 46. Considering that the cut-off score indicating elevated risk for autism is 54, this result places the child in the low-likelihood category, suggesting that there is currently no strong clinical indication of autism. 

## 4. Discussion

This paper presents a detailed characterization of the cognitive, speech–language, motor, sensory, adaptive, and socioemotional profile of a 4.5-year-old girl with Tetrasomy X. Given the heterogeneity and limited consistency of previous findings regarding the development of children with this chromosomal anomaly, the aim of this study was to further clarify the neurodevelopmental profiles of affected individuals, emphasizing the specificity of each case, in which developmental trajectories are shaped by the interplay of genetic and environmental factors.

Previous literature indicates that Tetrasomy X is associated with varying degrees of developmental delays and neurocognitive deficits. Regarding motor development, our assessment revealed that the child experienced difficulties from an early age, which is consistent with prior reports of low muscle tone and poor sensorimotor coordination, which contribute to delayed walking and lagging motor skills in girls with this condition [17,18,19]. Current evaluation placed her motor functioning within the low range of adaptive functioning, corresponding to a mild deficit. She demonstrated challenges in both gross motor skills and fine motor skills, and these findings align with previous studies reporting deficits in visual perception and visuomotor integration in girls with Tetrasomy X [14].

Assessment of the child’s cognitive functioning revealed an IQ score of 80, placing her in the borderline to low-average range of intellectual functioning. This result indicates that her general cognitive abilities are below age expectations but do not meet criteria for intellectual disability. The DQ was 77, reflecting a mild developmental delay and slower progress in achieving age-appropriate milestones across domains including motor skills, language, and cognitive functioning. Taken together, these findings indicate borderline intellectual and developmental functioning with mild delays. This profile contrasts with earlier reports suggesting that Tetrasomy X is generally associated with intellectual disability [5,8,20,21,22,23,24], and aligns more closely with recent evidence indicating that individuals with this karyotype can exhibit normal or near-normal cognitive functioning [14]. These findings underscore the phenotypic variability in cognitive outcomes among girls with Tetrasomy X and highlight the importance of individualized assessment and intervention planning rather than assuming uniform intellectual impairment.

Assessment of the child’s language development revealed marked delays, consistent with previous reports indicating that individuals with Tetrasomy X often present with language impairment [28]. The current evaluation demonstrated an uneven language profile. Receptive language abilities were relatively preserved for single words and basic concepts, indicating age-appropriate passive vocabulary. However, comprehension of more complex questions and multi-step instructions corresponded to the developmental level of a three-year-old, reflecting limitations in higher-order receptive language skills. These results align with prior studies suggesting that receptive language can also be compromised in this population [57,58]. Expressive language was considerably more affected, corresponding to a two-year developmental equivalent. Speech intelligibility was markedly reduced due to combined resonance and phonatory disturbances, alongside generally imprecise articulation. Articulatory precision was limited, with vowel shortening and other distortions significantly reducing overall intelligibility. Certain phonemes, including nasals, palatal approximants, and dental plosives, were relatively preserved, whereas others were consistently impaired or omitted. Further insight into her communication profile indicated that her overall communication abilities are slightly below age expectations, while her social–pragmatic competencies are stronger than would be expected based on her linguistic performance. Therefore, her primary difficulties are predominantly linguistic in nature and cannot be attributed to ASD. Adaptive communication skills, encompassing receptive, expressive, and written language, fell within the moderately low range, reflecting the functional impact of her speech and language delays. This profile is consistent with previous observations in individuals with Tetrasomy X [14,27,32,34] and aligns with findings across SCAs, where speech and language disorders are a core feature [6,11,13,14,27,28,29,30,31,32,33,34]. Taken together, these findings indicate a pronounced receptive–expressive gap, with relatively preserved comprehension and severely impaired expressive abilities, compounded by articulatory and phonatory difficulties. This pattern underscores the importance of early, targeted speech–language intervention focusing on expressive language development, phonological accuracy, and overall intelligibility, while leveraging her stronger receptive skills as a foundation for therapy.

Socioemotional assessment indicated that her maladaptive behavior profile (encompassing both internalizing and externalizing behaviors) falls within the average range. These findings indicate the absence of clinically significant levels of behavioral difficulties or emotional dysregulation. Notably, this profile contrasts with previous reports suggesting that some children with Tetrasomy X may exhibit heightened aggression or emotional lability [14,33].

To our knowledge, no prior studies have systematically examined sensory processing in children with Tetrasomy X. In the present case, evaluation revealed marked deviations from typical sensory development, with scores consistently falling between −1 SD and −2 SD across quadrant, sensory, and behavioral measures. This pattern indicates a reduced frequency of sensory-related behaviors and reflects global under-responsiveness to sensory input. Specifically, low scores in the Seeking, Avoiding, Sensitivity, and Registration quadrants suggest limited motivation to engage with sensory stimuli, reduced avoidance of potentially unpleasant input, diminished detection of sensory cues, and a tendency to miss or under-respond to environmental stimuli. Auditory, visual, movement, and body position processing were particularly affected, whereas touch and oral processing were somewhat less impacted but still fell below typical levels. Behavioral measures further indicated functional implications of sensory under-responsiveness, including reduced attention, difficulties with conduct regulation, and lower socioemotional engagement. Nevertheless, these results should be interpreted with caution. Although the overall profile is consistent with sensory under-responsiveness, it is important to consider that this pattern may partly reflect the child’s global psychomotor and developmental delays. Closer examination of individual items and parental comments suggests that her apparent low responsiveness may, at least in part, be secondary to difficulties with motor skills and speech–language abilities, which constrain her ability to respond effectively to auditory and visual stimuli as well as those involving movement and body position. These findings highlight a pattern of sensory under-responsiveness that may contribute to broader developmental challenges, affecting engagement, learning, emotional regulation, and social participation. Documenting sensory processing profiles in this population is novel and underscores the need for future research to investigate sensory characteristics systematically. Such studies could inform tailored interventions to optimize learning and adaptive functioning in children with Tetrasomy X.

To our knowledge, no previous studies have systematically examined adaptive functioning in children with Tetrasomy X. Assessment revealed that the child’s overall adaptive functioning falls within the moderately low range. However, domain-specific scores demonstrated marked variability. Daily Living Skills emerged as a relative strength, falling within the adequate range, indicating that the child demonstrates a degree of independence in managing personal care, household responsibilities, and basic community activities, reflecting well-developed self-care abilities. In contrast, Socialization was classified within the moderately low range; nonetheless, this domain represents a relative strength compared to her language abilities, suggesting that her social competence is partially constrained by linguistic deficits rather than intrinsic social difficulties. Weaknesses were most pronounced in the Motor Skills domain, highlighting the heterogeneous nature of functional development in Tetrasomy X. These findings suggest that strengths in specific domains, may partially mitigate weaknesses observed in other areas. These findings suggest that while global adaptive functioning may appear limited, targeted interventions that leverage her relative strengths could enhance overall independence and quality of life. Additionally, although some studies have suggested an association between SCAs and ASD [40], no such comorbidities were observed in this case. The child did not present with other neurodevelopmental disorders, indicating a relatively isolated profile of motor, cognitive, and speech–language difficulties within the context of Tetrasomy X.

## 5. Strengths and Limitations

This study offers a rare and comprehensive early developmental characterization of Tetrasomy X, combining detailed cognitive, speech–language, motor, sensory, and adaptive assessments. The use of multiple standardized and culturally adapted tools provides a multidimensional view of the phenotype and highlights previously underexplored features. However, as a single-case report, the findings can not be generalized beyond the present case and should be interpreted with caution. The cross-sectional design further limits conclusions regarding developmental trajectories, long-term outcomes, and the stability of observed strengths and weaknesses over time. Longitudinal follow-up would be essential to better understand the child’s developmental course, the evolution of the phenotype, and the impact of early interventions. Despite these constraints, the study adds valuable insight into the heterogeneous presentation of Tetrasomy X and underscores the importance of individualized, multidisciplinary evaluation.

## 6. Conclusions

This case contributes to the limited body of literature on Tetrasomy X by providing an early, detailed neurodevelopmental characterization, underscoring the marked intra-individual variability typical of this condition. The findings highlight the need for systematic, multidisciplinary evaluations to refine phenotype delineation and inform best clinical practices. Continued longitudinal and interventional research is essential to clarify developmental trajectories and optimize individualized management strategies.

## Figures and Tables

**Table 1:** Types of articulation errors.

	Correct	Types of Articulation Errors			
		Mild Distortions	Severe Distortions	Substitutions	Omissions
Vowels	/a/, /u/	/e/, /o/	/i/		
Plosives	/p/, /t/	/b/, /d/		/k/, /g/	
Affricates				/ts/, /dʑ/,	/tɕ/, /tʃ/, /dʒ/
Fricatives				/s/, /ʂ/, f/, /v/	/z/, /ʑ/, /h/
Aproximants	/j/				
Nasals	/m/, /n/, /ɲ/				
Laterals					/ɬ/, /ɮ/
Trill					/r/

## Data Availability

The original contributions presented in this study are included in the article/Supplementary Materials. Further inquiries can be directed to the corresponding author.

## Supplementary Materials

The following supporting information can be downloaded at: https://www.mdpi.com/article/10.3390/pediatric18020040/s1, Supplementary Material S1: Detailed description of administered instruments.

## Abbreviations

The following abbreviations are used in this manuscript:

SCA	Sex chromosome aneuploidy
ASD	Autism spectrum disorder
TEOAEs	Transient Evoked Otoacoustic Emissions
BERA	Brainstem Evoked Response Audiometry
DMSA	Dimercaptosuccinic acid scan
IEPSP	Institute for Experimental Phonetics and Speech Pathology
KSAFA	Kostić’s Selective Auditory Filter Amplifier
NBBS	New Belgrade Binet–Simon Scale
RTČ-P	Čuturić Developmental Test
PPVT-III-HR	Peabody Picture Vocabulary Test, third edition—Croatian version
NRDLS-SR	New Reynell Developmental Language Scales, Serbian version
GAT	Global Articulation Test
CCC-2	Children’s Communication Checklist—Second Edition
SLI	Specific language impairment
GCC	General Communication Composite
SIDC	Social Interaction Deviance Composite
SP-2	Sensory Profile 2
VABS-II	Vineland Adaptive Behavior Scales, second edition: parent/caregiver rating form
GARS-3	Gilliam Autism Rating Scale, third edition
AI	Autism Index
