# Supplementary material for: Neurodevelopmental Profile of a 4.5-Year-Old Girl with Tetrasomy X
Source: Pediatr Rep. 2026 Mar 9;18(2):40. doi: 10.3390/pediatric18020040 (PMC13010689; doi:10.3390/pediatric18020040)
Supplement: Supplementary file 1 [file pediatrrep-18-00040-s001.zip › pediatrrep-4143501-supplementary.pdf]

Detailed description of administered instruments

The New Belgrade Binet-Simon Scale (NBBS) [48] is a standardized intelligence assessment tool designed for children aged from 3 years and 10 months to 15 years. It is a revised and culturally adapted version of the original Binet-Simon scale, developed by the Institute of Psychology in Belgrade to account for the linguistic, educational, and cultural characteristics of the population in Serbia and the surrounding region. The NBBS comprises developmentally graded tasks that progressively increase in complexity and cover multiple cognitive domains, including verbal comprehension, abstract reasoning, memory, and problem-solving. Test items are organized according to mental age levels, following the classical Binet-Simon approach. The scale provides results in terms of mental age (MA) and intelligence quotient (IQ), calculated by comparing a child's mental age with their chronological age. It is widely used in clinical, educational, and research settings to identify intellectual strengths and weaknesses, support differential diagnosis, and guide intervention planning.

The Čuturić Developmental Test (RTČ-P) [49] is a standardized developmental screening instrument designed to assess psychomotor and cognitive development in children from birth to 7 years of age. It is widely used in Serbia and neighboring countries for the early identification of developmental delays. The test evaluates performance across multiple developmental domains, including motor skills, oculomotor coordination, communication, socialization, and cognitive abilities. Tasks are structured according to age levels, allowing for the assessment of whether a child's developmental progress aligns with normative expectations for their chronological age.

The Vineland Adaptive Behavior Scales, second edition: parent/caregiver rating form (VABS-II) [50] is a standardized instrument designed to assess adaptive behavior by measuring an individual's capacity for personal and social functioning across a range of daily life activities. The scale comprises four primary domains that evaluate developmental progress: communication, socialization, and daily living skills (assessed from birth to age 90) and motor skills (assessed from birth to age 7). Each domain is further divided into subdomains targeting specific skill sets. For instance, the communication domain includes receptive, expressive, and written communication; daily living skills are subdivided into personal, domestic, and community skills; socialization encompasses interpersonal relationships, play and leisure, and coping skills; and motor skills are assessed through gross and fine motor subdomains. Each subdomain contains targeted items that measure specific aspects of adaptive functioning.

The Peabody Picture Vocabulary Test, third edition – Croatian version (PPVT-III-HR) [51] was used to assess receptive vocabulary. During the assessment, children are shown sets of four images corresponding to each target word. The task required the child to identify the image that matched the word spoken by the examiner by pointing to it. The test started with items considered developmentally appropriate for the child's age and continued until the child made eight or more errors within a set. Results were reported as standardized scores.

New Reynell Developmental Language Scales, Serbian version (NRDLS-SR) [52] was used to assess language abilities. This instrument comprises two components: language comprehension and language production, each containing 72 items. The comprehension section evaluates multiple aspects of language development, including understanding of single words, relationships between two objects, verbs, clausal constituents, verb morphology, pronouns, complex grammar, and inferencing. The production section assesses the use of nouns, relational expressions, verbs, sentence construction, verb morphology, complex sentences, and grammaticality judgment. Results are reported as raw scores for each subcategory, which are subsequently converted into standard scores and age-equivalent scores for both comprehension and production.

The Global Articulation Test (GAT) [53] was used to assess articulation abilities. This test evaluates the pronunciation of speech sounds in Serbian. Since the Serbian language contains 30 phonemes, the test comprises 30 words, each of which the examinee is asked to repeat after the examiner. Each phoneme is scored as correctly produced, distorted (mild, moderate, or severe), substituted with another sound, or omitted entirely.

The Sensory Profile 2 (SP-2) [54] is a standardized instrument used to evaluate children's sensory processing patterns. Its primary purpose is to determine how a child's sensory processing affects daily functioning across various settings, including home, school, and the broader community. The assessment is completed by a caregiver or parent and consists of 86 items rated on a 5-point Likert scale. Each subscale score reflects the frequency of sensory processing behaviors, with higher scores indicating more frequent behaviors and lower scores indicating less frequent behaviors. Results are reported across multiple dimensions, including sensory systems, behavioral responses, and overall sensory processing patterns, and are interpreted according to Dunn's sensory processing framework.

The Gilliam Autism Rating Scale, third edition (GARS-3) [55] was used to assess the presence of autistic symptoms. This parent- and caregiver-administered questionnaire contains six subscales: repetitive/restrictive behaviors, social interaction, social communication, emotional reactions, cognitive style, and non-functional speech. The full questionnaire contains 58 items. Responses are rated on a four-point Likert-type scale ranging from 0 = never observed to 3 = frequently observed. Raw scores were converted into standard scores for each subscale. The sum of these standard scores constitutes the Autism Index (AI), indicating both the probability of the presence of autism and the severity of symptoms: an AI score of ≤54 suggests autism is unlikely, 55–70 indicates likely presence with severity level 1, 71–100 indicates very likely presence with severity level 2, and scores ≥101 indicate severity level 3.

The Children's Communication Checklist – Second Edition (CCC-2) [56] is a standardized assessment tool designed to evaluate communication skills in children aged 4 to 16 years. It was developed to identify children who may exhibit pragmatic language impairments, including those associated with ASD, specific language impairment (SLI), or other developmental conditions. The CCC-2 is completed by a parent, caregiver, or teacher and comprises 70 items organized into 10 scales: speech, syntax, semantics, coherence, inappropriate initiation, stereotyped language, use of context, nonverbal communication, social relations, and interests. These scales are combined to produce a General Communication Composite (GCC), providing an overall measure of communication ability, and a Social Interaction Deviance Composite (SIDC), which helps differentiate between children with general language impairments and those with more pronounced pragmatic difficulties, such as those observed in ASD.
